# Efficacy of education delivery through multimedia and text messaging on the psychological parameters of patients scheduled for coronary angiography: a single-blind randomized controlled clinical trial

**DOI:** 10.1186/s12872-020-01820-7

**Published:** 2021-01-04

**Authors:** Camellia Torabizadeh, Sara Rousta, Sakineh Gholamzadeh, Javad Kojouri, Kavoos Jamali, Mohammad Mahdi Parvizi

**Affiliations:** 1 School of Nursing and Midwifery, Shiraz University of Medical Sciences, Shiraz, Iran; 2 Education Development Center, Shiraz University of Medical Sciences, Shiraz, Iran; 3 Kowsar Hospital, Shiraz University of Medical Sciences, Shiraz, Iran; 4 Molecular Dermatology Research Center, Shiraz University of Medical Sciences, Shiraz, Iran; 5 Clinical Education Research Center, Shiraz University of Medical Sciences, Shiraz, Iran

**Keywords:** Coronary angiography, Text messaging, Multimedia, Mental disorder, Patient education

## Abstract

**Background:**

Angiography is a highly effective invasive method for diagnosing coronary artery diseases but can lead to certain psychological problems such as stress, anxiety, and depression. This study aimed to compare the effects of education delivery through multimedia DVD content or text messaging in comparison with conventional printed pamphlets on the psychological parameters of patients scheduled for angiography.

**Methods:**

This study was a randomized controlled clinical trial. We used the convenience sampling method to select 120 patients who met the inclusion criteria among individuals who were scheduled for elective coronary angiography. The patients were randomly divided into three groups through the block randomization method. The necessary educational tips for before, during, and after the angiography procedure were delivered to the patients in interventional group 1 (n = 40) and interventional group 2 (n = 40) through text messages and a multimedia DVD, respectively. The control group received routine hospital education through pamphlets besides the opportunity for verbal discussions with nurses. All educational content was delivered to the patients four days before the scheduled day of angiography. The DASS-21 questionnaire, consisting of the three domains of stress, anxiety, and depression, was used to collect the data. The questionnaire was administered at the time of delivering the educational content as the pretest evaluation and 30 min after the angiography procedure as the posttest evaluation. SPSS software, version 18, was used for statistical analysis.

**Results:**

There was no significant difference among the three groups of patients in terms of pretest DASS-21 scores. Conversely, the mean posttest scores in all DASS-21 domains were significantly lower among the patients receiving education via the multimedia DVD or text messaging in comparison with the control group (*P* < 0.001). However, there were no statistically significant differences between the mean scores in all domains between the participants in the DVD and text messaging groups.

**Conclusion:**

It seems that both DVDs and text messaging are more effective than conventional pamphlets in controlling the anxiety, stress, and stress of patients scheduled for elective coronary artery angiography.

*Iranian Registry of Clinical Trials*: IRCT registration number: IRCT2015030121283N1, Registration date: 2015-10-05, 1394/07/13

## Background

Coronary angiography is a standard and highly reliable diagnostic method that helps the physician in managing a patient with coronary artery disease [1]. Evidence shows that patients scheduled for coronary angiography often experience anxiety, stress, and depression [2, 3]. Furthermore, having sufficient information about this invasive procedure can help control vital signs including systolic blood pressure, diastolic blood pressure, and the heart rate in patients awaiting coronary angiography [4, 5]. According to the literature, mobile phone-based patient education can decrease the anxiety of patients scheduled for coronary angiography [6, 7]. Selecting useful and applicable methods to educate patients is very important because several barriers exist in this area, including physical and psychological problems like a low capacity to learn, an inability to comprehend the information, or a lack of motivation [8].

Most of the educational materials for patients in hospitals and health centers in Iran are based on educational pamphlets made available in the Persian language. These pamphlets do not take into account the age, education level, social, economic, and cultural factors, and the native language or dialect of the patient [9], undermining their efficacy [10]. A review study revealed that nowadays, traditional patient education approaches cannot meet the substantial needs in patient–doctor and patient–nurse relationships. Hence, many experts try to modify and improve the traditional methods of education and also make use of novel means of delivering patient education [11].

Education delivery via novel technologies leads to much greater satisfaction in comparison with the traditional methods of lecturing and presenting information through pamphlets [12]. Web-based learning and educational Digital Versatile Discs (DVDs) are two popular self-study methods in electronic learning. Learning via multimedia engages the learner's various senses and allows for the simultaneous use of visual, auditory, and textual teaching material; it also helps the patient learn what they need to learn better and face the actual situations more easily [13, 14].

The Short Message Service (SMS) is an especially popular mobile phone service due to the convenience, flexibility, and possibility of establishing effective communication. In the United States and Europe, manual devices for patient monitoring and instruction delivery have become extremely popular and mobile phones have become convenient and readily available devices for patient use [15, 16]. In addition, text messaging represents a cost-efficient opportunity to transfer medical information to patients [17].

In recent years, the use of virtual media in patient education has become a popular area of research, with several studies having been conducted to evaluate the efficacy of several aspects of the related educational methods. To our knowledge, there are no prior studies on the efficacy of patient education through text messaging or DVD multimedia content in comparison with conventional pamphlets (as the control group) on anxiety, stress, and depression among patients scheduled for coronary artery angiography. Therefore, in this three-arm randomized controlled clinical trial, we tried to compare the efficacy of patient education delivery via two different modern electronic-based methods (DVDs and text messaging) with a paper-based method.

## Methods

### Study design

This was a single-blind randomized controlled clinical trial that was conducted with a pretest–posttest design from April to October 2015. The study population consisted of patients who were scheduled for coronary artery angiography in selected hospitals affiliated to Shiraz University of Medical Sciences, Shiraz, Iran, namely Namazi Hospital, Faghihi Hospital, Kowsar Heart Hospital, and Al-Zahra Heart Hospital. Two cardiologists who performed coronary artery angiography in the selected hospitals were involved in the study. The investigation conformed with the principles outlined in the Declaration of Helsinki. The study protocol was explained to all participants and written informed consent was obtained. Furthermore, the participants were free to withdraw from the study at any stage. The Ethics Committee of Shiraz University of Medical Sciences approved the protocol of this study (Code: CT-93–7343). In addition, the protocol of this project was registered in the Iranian Registry of Clinical Trials (IRCT) website with code IRCT2015030121283N1 (https://www.irct.ir/trial/18683).

### Sample size and study population

The minimum sample size of 40 per group was estimated using the means comparison formula based on mean anxiety scores, on the basis of an alpha = 0.05 significance level, a power of 80%, and anxiety scores of 9.6 ± 2.22 in the intervention group and 11.8 ± 4.28 in the control group reported by Hanifi et al. [12]. By considering the loss to follow-up of patients, especially because of withdrawal from the study or coronary angiography intervention cancellation, two to three extra patients were enrolled in each group to the study.

### Inclusion and exclusion criteria

The inclusion criteria were as follows: (1) the scheduled coronary artery angiography would be the first experience of the patient with the procedure; (2) an age range of 25–75 years (the common age range in adults for coronary angiography); (3) lack of a job related to medical care and treatment; (4) ability to receive text messages via cell phone and to use a computer for watching multimedia content stored on a DVD; (5) literacy (defined as being able to read and write with completion of a minimum of five years of elementary school); and (6) a minimum of four days being left until the angiography appointment. The exclusion criteria were as follows: (1) experience of another invasive diagnostic procedure, such as transesophageal echocardiography or prior angiography; (2) a low level of consciousness or known case of a psychological disorder; and (3) a positive history of psychiatric medication use.

### Randomization and blinding

The Random Allocation Software Ink (Version 1.0, May 2004) was applied to create a randomization table, considering a block size of six including the equal times of each intervention of the study in each block. Therefore, the patients were randomly assigned and allocated to three groups, namely the DVD (n = 40), text messaging (n = 40), and control (n = 40) groups, coding using the letters A, B, and C, respectively, thereby blinding the staff who evaluated the patients as well as the statistical analyzer.

### Data collection and questionnaire

The data collection instruments included a researcher-made demographics questionnaire and Depression Anxiety Stress Scale-21 (DASS-21). This scale is used for measuring depression, anxiety, and stress using a total of 21 items. The instrument is designed based on the four-point Likert scale scored with 0 for never, 1 for sometimes, 2 for often, and 3 for almost always. In this regard, stress was evaluated through items 1, 6, 8, 11, 12, 14, and 18, anxiety through items 2, 4, 7, 9, 15, 19, and 20, and depression through items 3, 5, 10, 13, 16, 17, and 21. For obtaining the total score in each domain, the scores of the mentioned items are summed up. Antony et al. (1998) used Cronbach's alpha coefficient for the internal consistency of the scale, reporting values of 87, 82, and 78 percent for the stress, anxiety, and depression domains, respectively. The overall reliability of the scale was found to be 93% [18]. The reliability of the Persian version of this questionnaire was ascertained in one study among 390 individuals from the general population of Tehran, Iran, with Cronbach's alpha coefficient value being reported at 0.85 for the depression domain, 0.85 for the anxiety domain, and 0.87 for the stress domain. Furthermore, the test–retest reliability reportedly revealed interclass correlation with absolute agreement between first and second evaluations with scales of 0.77, 0.89, and 0.85 for the depression, anxiety, and stress domains. The study also revealed acceptable discriminate validity in all domains of the questionnaire [19].

### Interventions and follow-ups

Overall, 120 patients who were scheduled for elective coronary artery angiography and met the inclusion criteria were enrolled in the study. According to the block randomization method, the patients were randomly divided into three groups: the text messaging group, the DVD group, and the control group. In the pretest stage, the patients were asked to complete the demographic questionnaire (age, gender, education, and marital status) and the DASS-21. During the four days leading to the appointed day of angiography, the patients in intervention group 1 were sent seven short text messages (via mobile phone) each day with information regarding the purpose and procedure of coronary angiography and what the patients should know and do in the peri-coronary artery angiography period. The patients in intervention group 2 received a DVD containing 15 min of educational multimedia content. The educational content of the text messages, the DVD, and the pamphlets were practically the same, except that the DVD also contained related pictures and animations. The control group simply received the conventional pre-operation hospital care, which consisted of basic verbal information and a pamphlet on what to do before angiography (e.g., refraining from food), how angiography is performed, and how the medication should be taken. The content validity of the text messages, DVD multimedia, and pamphlets was approved by a six-member team including two cardiologists, two nurses each with a Ph.D. degree, and one general physician with a master’s degree in medical education. The contents of the multimedia DVD were produced using Adobe Flash Professional CS6 2015.

Thirty minutes after the completion of coronary artery angiography, the questionnaire was filled out again by the patients to determine the posttest scores. Beforehand, the researcher asked the patients whether they received and read the messages or watched the DVD content; individuals who did not take up the patient education were excluded at this point in the study.

### Statistical analysis

The data were analyzed using SPSS, version 18. The Kolmogroph-Smirnoff test was used to evaluate the data distribution. The chi-squared test and Fisher's exact test were used to compare the three groups in terms of their demographic variables; analysis of variance (ANOVA) was used with a post hoc test (Tukey HSD) to compare the patients’ anxiety, stress, and depression scores. The paired *t*-test was used to compare the scores of the DASS-21 questionnaire before and after the intervention separately in each group. A *P*-value of less than 0.05 was considered significant.

## Results

**Fig. 1:**
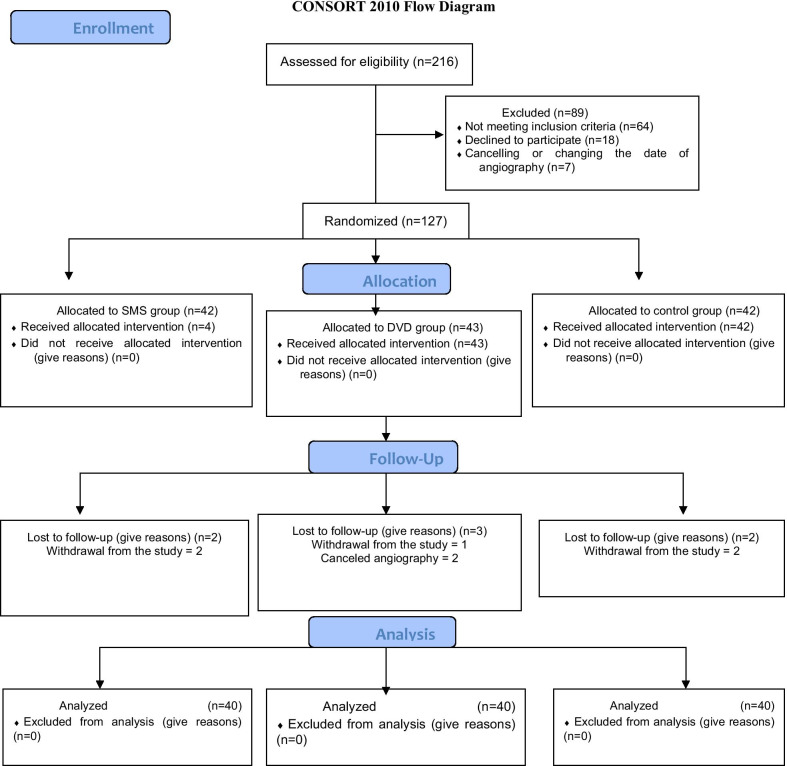
CONSORT flow diagram of the effects of education delivery through multimedia and short text messaging on the psychological parameters of patients scheduled for coronary angiography

The CONSORT chart of this study is shown in Fig. 1. The mean patient age was 55 ± 9.65 years, and 55% of the participants were male. The results showed no significant difference between the patients in the intervention groups and the control group in terms of marital status (*P* = 0.87), education (*P* = 0.51), gender (*P* = 0.90), and age (*P* = 0.78) (Table 1).

**Table 1:** Demographic characteristics of the patients scheduled for coronary angiography in the short text messaging, DVD, and control groups

Qualitative variables	SMS^a^ (n = 40)	DVD^b^ (n = 40)	Control (n = 40)	Total sample (%)	*P*-value
Sex no. (%)					
Female	19 (5.47)	18 (45.00)	17 (5.42)	54 (45.00)	0.90
Male	21 (5.52)	22 (55.00)	23 (5.57)	66 (55.00)	
Marital status no. (%)					
Married	36 (90.00)	36 (90.00)	35 (5.87)	107 (89.20)	0.87
Single	1 (5.20)	0 (0.00)	1 (5.20)	2 (1.70)	
Widowed	3 (5.70)	4 (10.00)	4 (10.00)	11(9.20)	
Educational level no. (%)					
Elementary school	13 (5.32)	15 (5.37)	16 (40.00)	34 (36.60)	0.51
Middle school	3 (5.70)	5 (5.12)	5 (5.12)	13 (10.80)	
High school	5 (5.12)	3 (5.70)	7 (5.17)	15 (12.50)	
Diploma	11 (5.27)	10 (25.00)	8 (20.00)	29 (24.20)	
Associate degree	3 (5.70)	4 (10.00)	3 (5.70)	10 (8.30)	
Bachelor’s degree and higher	5 (5.12)	3 (5.70)	1 (5.20)	9 (7.00)	
Age					
Mean ± SD	54.92 ± 9.6	56 ± 9.06	54.55 ± 10.39	0.78	

^a^Short message service

^b^Multimedia content is stored on a DVD

According to ANOVA conducted on the patients’ data before the interventions, there were no significant differences among the patients in the mean pretest scores of all DASS-21 domains. By the end of the study, the results showed significant drops in the mean score of anxiety (*P* < 0.001), stress (*P* < 0.001), and depression (*P* < 0.001) among patients in both intervention groups. However, in the patients of the control group, there was a statistically significant difference between their pretest and posttest mean anxiety score (*P* < 0.001), but this was not true for their depression (*P* = 0.15) and stress scores (*P* = 0.13). These results are summarized in Table 2. According to the post hoc test, the posttest depression domain score of the control group significantly differed with that of the text messaging (*P* = 0.019) and DVD (*P* < 0.001) groups. Such significant posttest differences between the control group and the text messaging and DVD intervention groups were also seen in the anxiety (*P* = 0.071 and *P* = 0.006, respectively) and stress (*P* < 0.001 and *P* < 0.001, respectively) domains.

**Table 2:** The pretest and posttest mean anxiety, stress, and depression scores in the patients scheduled for coronary angiography in short text messaging, DVD, and control groups

Variables	Time of evaluation	SMS^a^ (n = 40)	DVD^b^ (n = 40)	Control (n = 40)	Pretest** *P*-value	Posttest** *P*-value
Anxiety	Pretest	8.35 ± 7.91	7.12 ± 8.20	7.15 ± 6.15	0.70	< 0.001***
	Posttest	3.45 ± 2.61	2.50 ± 0.00	2.42 ± 1.90		
	*P*-value*	< 0.001	0.001	< 0.001		
Stress	Pretest	7.77 ± 4.50	7.62 ± 3.02	6.22 ± 3.97	0.14	< 0.001***
	Posttest	3.27 ± 3.70	4.07 ± 2.04	7.55 ± 4.46		
	*P*-value	< 0.001	< 0.001	0.13		
Depression	Pretest	5.90 ± 4.96	4.87 ± 5.35	6.35 ± 5.59	0.44	< 0.001***
	Posttest	5.10 ± 5.36	3.55 ± 4.73	5.85 ± 4.52		
	*P*-value	< 0.001	< 0.001	0.15		

Tukey HSD was used. According to the post hoc test, the posttest depression domain score of the control group significantly differed with that of the text messaging (*P* = 0.019) and DVD (*P* < 0.001) groups. Such significant posttest differences between the control group and the text messaging and DVD intervention groups were also seen in the anxiety (*P* = 0.071 and *P* = 0.006, respectively) and stress (*P* < 0.001 and *P* < 0.001, respectively) domains

*Paired *t*-test

**ANOVA test

***For the post hoc test

^a^Short message service

^b^Multimedia content stored on a DVD

A comparison between the mean change in scores between the text message group and the control group showed that there was a significant difference between their pretest and posttest scores with regard to stress (*P* < 0.001), but not with regard to depression (*P* = 0.77) and anxiety (*P* = 0.07). In this regard, a comparison of the mean change in scores between the DVD group and the control group showed that the use of the DVD led to a significant reduction in anxiety (*P* = 0.006) and stress scores (*P* < 0.001), but not in depression scores (*P* = 0.09). However, in the control group, the only significant difference was a reduction of the mean anxiety score (*P* < 0.001) (Table 3).

**Table 3:** Comparison of the changes in the means scores of anxiety, stress, and depression in the patients scheduled for coronary angiography in the short text messaging, DVD, and control groups

Groups	Control (n = 40)		SMS^a^ (n = 40)		DVD^b^ (n = 40)	
	Mean difference	*P*-value	Mean difference	*P*-value	Mean difference	*P*-value
Anxiety						
SMS^a^	− 1.02	0.07	–	–	0.42	0.62
DVD^b^	− 1.45	0.006	− 0.42	0.62	–	
Stress						
SMS^a^	− 4.27	< 0.001	–	–	− 0.80	0.57
DVD^b^	− 3.47	< 0.001	0.80	0.57	–	–
Depression						
SMS^a^	0.75	0.77	–	–	1.55	0.33
DVD^b^	− 2.3	0.09	− 1.55	0.33	–	–

^a^Short message service

^b^Multimedia content stored on a DVD

There was no significant difference between the DVD and text message groups in the changes in their mean anxiety (*P* = 0.62), stress (*P* = 0.57), and depression scores (*P* = 0.33) (Table 3).

## Discussion

The results of our study revealed that there were significant decreases in the scores of anxiety, stress, and depression among the patients in the intervention groups of the study. In this regard, education through multimedia and short text messages were both effective in reducing the stress, anxiety, and depression levels of patients in the intervention groups. However, in the control group, verbal discussion and pamphlets were only effective in reducing the mean anxiety score of the patients. This reduction might be due to face-to-face contact with hospital nurses, who could immediately answer their questions about their disease, which could influence the rate of such psychological reactions as anxiety in these patients. Peterson et al. pointed out that educational and social interventions were effective ways of decreasing the level of anxiety on the day of procedure among patients awaiting cardiac catheterization [20]. The study of Najafi-Kalyani et al*.* revealed that the patients’ low level of awareness about how angiography is going to be done and the necessary care before and immediately after the procedure can lead to the patients’ sadness and dissatisfaction; therefore, the patients would not tolerate adhering to the necessary care, which may eventuate in an increase in vascular complications [21].

According to the literature, the most important advantages of education through multimedia content are the lack of a need for a trainer, time-efficiency, ease of use, and affordability [22]. The use of multimedia content and smartphone-based education has been considered in other aspects of the education of patients with cardiac problems. In this regard, Wang et al*.* revealed that a multimedia exercise training program could increase the patients’ walking distance and improve the patients’ heart rate. In addition, they found this kind of education could increase the self-efficacy of cardiac surgery patients, even in the month after discharge from the hospital [23]. Moreover, a number of smartphone-based applications have been developed for educating patients with coronary artery diseases in recent years [24].

In the present study, the multimedia DVD was made available to the patients a few days before the day of coronary artery angiography. The absence of difficult medical terms and the combination of sound with simple and interesting slides and animations allowed the patients to experience multimedia learning. These results are in line with those of previous studies, which revealed that educating patients through video and multimedia content decreased preoperative anxiety and stress [25–29].

The text messaging approach was highly effective as it allowed the patients to receive the required information via their personal mobile phone device. Moreover, since the messages were sent at specified times, the participants were motivated to read them immediately and to save them systematically; therefore, they learned more about coronary artery angiography, corrected their misconceptions, and consequently experienced less tension before the procedure [28, 30].

Based on the literature, sending short text messages can play an important role in patient education. The study of Kumar et al. demonstrated that short text messages could prove extremely effective for educational purposes; for example, learning via short text messages is highly flexible and is resistant to power cuts and oblivion [28, 30]. In addition, Park et al*.* claimed that education through short text messages was more effective than a smartphone-based application in implementing a secondary prevention program among patients with coronary artery diseases. According to that study, this method of education resulted in improved clinical outcomes and patient lifestyle [31].

A comparison between the mean change in depression scores of patients in the text message and DVD groups on one hand and the control group on the other showed that the interventions did not lead to a significant reduction in depression scores compared with the control. Accordingly, the researchers suggest that evaluation of the psychological problem of the patients due to coronary artery angiography would be more acceptable if they fill out the questionnaires at a more distant time from the end of the procedure, when he/she has had more time to come to terms with the events. Furthermore, it is suggested that the use of social virtual networks and multimedia messaging services, which allow transferring interesting and spectacular pictures and videos along with text messages, could help boost patient motivation.

In the present study, a comparison between the DVD multimedia and text message groups' mean scores for the psychological parameters of anxiety, stress, and depression showed that there was no significant difference between these two groups. In other words, both educational methods were effective in reducing the anxiety, depression, and stress of the patients. It seems that the use of text, video, and audio in delivering education to patients is effective in improving these psychological parameters before coronary artery angiography [32, 33].

It is possible that the similarity between the effects of the multimedia and text messages on the participants' psychological parameters was because of the advantages of each method and their appeal to the patients. The evident advantages of text messaging are ease of use and the possibility of sending large numbers of texts in a very short time, while the obvious advantage of multimedia is the possibility of using both audio and video content. Among the shared advantages of the two methods are their low cost, repeatability of education as desired by the learner, lack of need for face-to-face interaction, and elimination of time and place limitations [34, 35]. Among the strong points of the study was the similarity of the contents presented through both methods of education and the possibility of repeatedly making use of the content for the patients.

There were some limitations to this study. Some of the participants did not have access to or were not able to use a computer or laptop and play the multimedia DVD at home and were, therefore, excluded from the study. Accordingly, it is possible that patients with low socioeconomic conditions were excluded from the study. In this regard, we suggest that patients should receive such educational content through virtual networks, which have become quite popular among the public. Furthermore, although the patients were given information about the objectives of the study and they were asked not to share information with other participants, there was a possibility of the participants sharing information or using other sources of information because the patients were not blinded to the intervention. In this regard, the authors suggest that multi-central studies be conducted with a high number of participants.

## Conclusion

The results of the present study showed that stress, anxiety, and depression affected most patients scheduled for coronary artery angiography; thus, there was a need for addressing this issue. According to our findings, informing patients about what they will experience in the catheterization laboratory through a multimedia DVD and short text messages can reduce the anxiety, stress, and depression of patients scheduled for coronary artery angiography. In the present study, both methods of education delivery were found to be effective in reducing these negative psychological parameters among the participants.

### Lay summary

The results of the present study showed that patient education via multimedia and text messaging was effective in reducing negative psychological parameters in patients after angiography. These interventions are recommended for patient education purposes and it is suggested that nursing students should be introduced to such methods of providing patient education and their advantages. These methods can also be used for on-the-job training for nurses’ problems.

## Data Availability

The datasets used and/or analyzed during the current study are available from the corresponding author on reasonable request.

## Abbreviations

DASS-21: Depression, anxiety and stress scale—21 items
DVD: Digital versatile discs
IRCT: Iranian registry of clinical trials
SMS: Short message service
SPSS: Statistical package for the social sciences
